# Specific amplifications and copy number decreases during human neural stem cells differentiation towards astrocytes, neurons and oligodendrocytes

**DOI:** 10.18632/oncotarget.15980

**Published:** 2017-03-07

**Authors:** Ulrike Fischer, Ella Kim, Andreas Keller, Eckart Meese

**Affiliations:** ^1^ Department of Human Genetics, Saarland University, Homburg/Saar, Germany; ^2^ Translational Neurooncology Research Group, Johannes Gutenberg University, Mainz, Germany; ^3^ Clinical Bioinformatics, Saarland University, Saarbrücken, Germany

**Keywords:** gene amplification, CDK4, MDM2, EGFR, astrocytes, Neuroscience

## Abstract

There is growing evidence that gene amplifications are an attribute of normal cells during development and differentiation. During neural progenitor cell differentiation half of the genome is involved in amplification process. To answer the question how specific amplifications occur at different stages and in different lineages of differentiation we analyzed the genes *CDK4*, *MDM2*, *EGFR*, *GINS2*, *GFAP*, *TP53*, *DDB1* and *MDM4* in human neural stem cells that were induced to differentiate towards astrocytes, neurons and oligodendrocytes. We found specific amplification pattern for each of the eight analyzed genes both in undifferentiated neural stem and progenitor cells and in cells that were induced for differentiation. Different amplification patterns were also found between adherently grown neural stem cells and cells that were grown as spheres. The most frequently amplified genes were *MDM2* and *CDK4* with the latter amplified in all three lineages at all analyzed stages. Amplification of the analyzed genes was also found in four glioma stem-like cells. The combined amplification data of stem cells and of tumor stem cells can help to define cell populations at the origin of the tumor. Furthermore, we detected a decrease of gene copies at specific differentiation stages most frequently for *MDM4*. This study shows specific amplification pattern in defined stem cell populations within specific time windows during differentiation processes indicating that amplifications occur in an orderly sequence during the differentiation of human neural stem and progenitor cells.

## INTRODUCTION

There is ample evidence for gene amplifications in human tumors [1, 2]. More recently, several studies reported gene amplification also in stem cells or progenitor cells during developmental processes and specifically during differentiation. As part of the embryonic development *ERBB2* gene amplification occurs in human trophoblast cells [3]. Recently, amplification of placental genes was reported in trophoblast giant cells [4]. We found a larger number of amplifications using array-CGH and fluorescence *in situ* hybridization during differentiation of human neural progenitor cells and mouse neural stem and progenitor cells [5, 6]. We also detected gene amplifications during the differentiation of human and mouse myoblasts towards muscle cells [7]. Amplifications during the differentiation process occur apparently only in small sub-population of the cells [5] making them difficult to detect especially in high throughput assays, which mostly analyze a large number of cells. Although the presence of amplifications as part of developmental process appears to be assured, the biological role of amplifications in this physiological process is less well established. As for many mutations, amplifications can be a driving force or a bystander for these processes. With only a few cells carrying amplifications, it is near to impossible to obtain evidence for functional relevance by determining the expression levels of the amplified genes within a cell population that mostly contains cells without gene amplification. Alternatively, amplifications that occur in an orchestrated way during specific cellular processes may be indicative of functional relevance as opposed to amplifications that occur randomly. Our abovementioned studies on the differentiation of human and mouse myoblasts towards muscle cells provided first evidence for ordered amplification events. Here, we set out to answer the question whether amplifications occur in an orderly sequence as part of the differentiation of human neural stem cells. To this end, we compared the sequence of amplification events during three different lineages of differentiation and ask for the specificity of an amplification pattern for each of these processes. In detail, we differentiated neural stem cells towards astrocytes, neurons and oligodendrocytes to investigate gene amplifications.

## RESULTS

**Figure 1:**
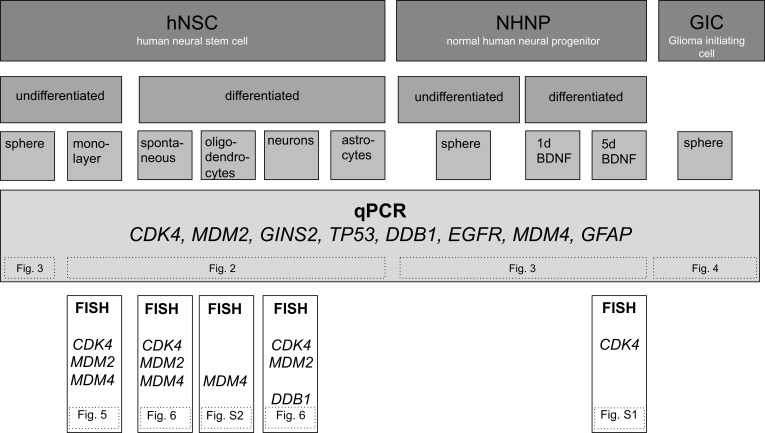
Overview of experimental design Graphic overview on used cells, differentiation induction, and techniques to analyze amplification. Reference to other figures, is given by numbers in dashed boxes.

An overview on experimental design is shown in Figure 1. To analyze amplifications during different lineages of differentiation we induced differentiation of adherent growing human neural stem cells (NSC; H9 hESC-derived; GIBCO) into oligodendrocytes, astrocytes, and neurons. In detail, NSC were grown as adherent cells on CELL Start^TM^ treated culture surface with EGF and bFGF for 24h in the following referred to as time point 0 h. Subsequently, NSC cells were induced to differentiate towards oligodendrocytes with Neurobasal® medium supplemented with B-27® Serum-Free Supplement, GlutaMAX™-I and T3 on polyornithine and laminin-coated culture dish. Differentiation towards neurons was induced by Neurobasal® medium supplemented with B-27® Serum-Free Supplement and GlutaMAX™-I on polyornithine- and laminin-coated culture dish. Differentiation towards astrocytes was induced by D-MEM supplemented with N-2, GlutaMAX™-I, and 1% FBS on Geltrex® matrix–coated culture dish. Spontaneous differentiation was induced by growth factor depletion. In each of the four assays DNA was isolated four times after 24 hours each (1-4 days). For all lineages of differentiation and all time points we determined the copy number of eight genes including *CDK4*, *MDM2*, *EGFR*, *GINS2*, *GFAP*, *TP53*, *DDB1* and *MDM4* all of which are known to localize to amplified genomic regions in neural progenitor cells during differentiation and to be amplified in human glioblastoma. The amplification was determined by qPCR analysis (TaqMan) in four replicates with the data analyzed by the software “copy caller“ (Applied Biosystems) as described previously [7, 8]. Mean calculated copy numbers for control DNA from blood lymphocytes revealed values in the range from 1.8 to 2.14 and were further regarded as normal diploid copy number. A decreased copy number was defined by values <1.8, an increased copy number by values > 2.2 and < 2.3 and an amplification by values > 2.3. Results of all experiments were summarized in Table 1.

**Table 1:** Results of copy number analysis in neural stem and progenitor cells

	CDK4	MDM2	GINS2	TP53	DDB1	EGFR	MDM4	GFAP
PB	2	2	2	2	2	2	2	2
NSC 0h	2,36	2,13	2,1	2,09	1,52	1,88	2,36	2,34
spontan 24h	3,28	2,4	2,49	2,31	2,18	2,09	1,88	2,08
spontan 48h	3,13	2,6	2,26	2,27	2,19	2,03	1,94	2,03
spontan 72h	2,71	2,55	2,25	2,27	2,18	2,1	1,86	2,03
spontan 96h	2,86	2,42	2,25	2,23	2,28	1,97	1,95	2,02
Oligo 24h	3,35	2,1	2,33	2,01	1,83	1,6	1,99	2,06
Oligo 48h	2,9	2,26	1,94	1,93	1,75	1,59	1,55	1,76
Oligo 72h	2,6	2,15	1,96	2,02	1,92	1,77	1,57	1,74
Oligo 96h	3,35	2,59	2,21	2,14	2,07	2,02	1,76	1,92
Neu 24h	3,19	2,48	2,33	2,27	2,25	1,82	1,95	2,02
Neu 48h	3,2	2,67	2,12	2,17	2,26	1,89	1,75	1,93
Neu 72h	2,56	2,51	2,1	2,08	2,02	1,91	1,62	1,82
Neu 96h	2,53	2,5	2,11	2,17	2,16	2,02	1,68	1,89
Astro 24h	3,11	2,73	2,25	2,29	2,3	2,16	1,74	1,96
Astro 48h	3,15	2,49	2,26	2,25	2,33	2,07	1,75	1,92
Astro 72h	2,61	2,76	2,18	1,88	2,22	2,07	1,68	1,92
Astro 96h	2,58	2,46	2,26	2,21	2,22	2,02	1,91	2,02
NSC sphere	3,21	2,06	2,58	2,23	2,15	1,94	2,36	2,22
NHNP sphere	3,1	2,33	2,49	2,45	2,62	2,53	2,08	2,32
BDNF 1d	2,63	2,18	2,33	2,26	2,48	2,22	1,85	1,98
BDNF 5d	2,94	2,29	2,41	2,35	2,59	2,5	1,98	2,08

Mean calculated copy numbers from four replicate experiments were defined as follows: copy number increase: 2.2-2.3 (light green), amplification: >2.3 (dark green), under- replication: < 1.8 (orange)

**Figure 2:**
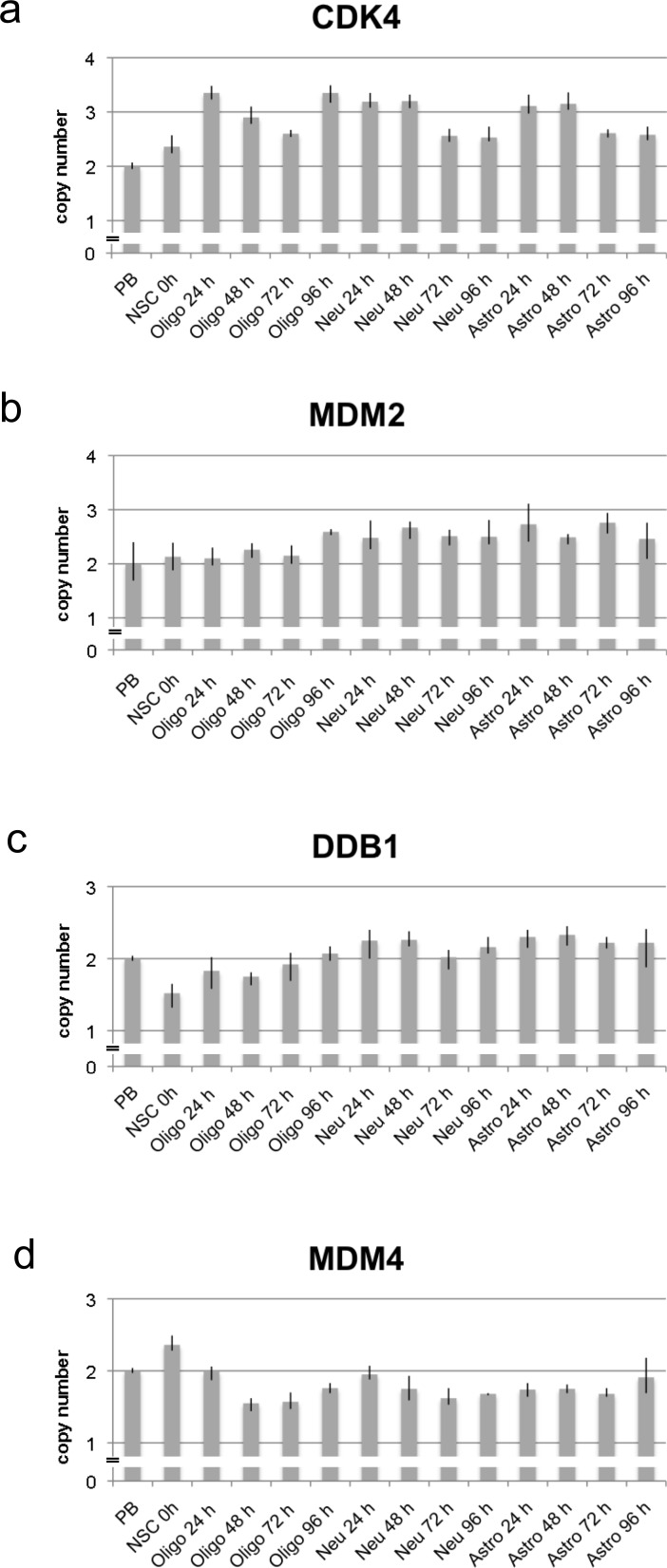
Copy number analysis of CDK4, MDM2, DDB1 and MDM4 using qPCR in neural stem cells and during differentiation Copy number of *CDK4, MDM2, DDB1* and *MDM4* was analyzed by qPCR using TaqMan copy number assays. *RNaseP was used* as reference gene in the TaqMan assays and DNA from normal blood lymphocytes (PB) served as standard for normal diploid copy number. Copy numbers are shown as mean from four technical replicates with vertical lines indicating the range. NSC cells (oh) served as undifferentiated control. Differentiation induced NSC cells were analyzed at four time points after differentiation induction. Differentiation induction was either towards oligodendrocytes (oligo), towards neurons (neu) and towards astrocytes (astro). *CDK4* was amplified with the highest copy number after 24 h and 96 h of differentiation towards oligodendrocytes and after 24 h and 48 h differentiation towards neurons and astrocytes **a**. *MDM2* was amplified with the highest copy number after differentiation towards neurons and astrocytes **b**. *DDB1* revealed a decreased copy number in undifferentiated NSCs and after 48 h differentiation towards oligodendrocytes whereas an increased copy number was detected after differentiation towards neurons and astrocytes **c**. *MDM4* was amplified only in undifferentiated NSCs whereas a decreased copy number was detected from 48 h differentiation towards oligodendrocytes and neurons and from 24 h differentiation towards astrocytes **d**.

As for *CDK4* the copy number was already amplified in undifferentiated NSCs (0 h) as compared to peripheral blood that was used as reference. We found a high copy number increase of *CDK4* 24 h after differentiation induction in all 4 differentiation protocols. In all protocols the *CDK4* copy number decreased at 72 h. For the last time point (96 h) we found divergent results with a strong increase of the copy number in differentiating oligodendrocytes, a moderate increase in the spontaneously differentiated cells, and a low copy number in differentiating astrocytes and neurons (Figure 2a and Table 1). *MDM2* that is localized in close vicinity to *CDK4* on chromosome 12, showed a different amplification pattern upon differentiation induction. Among the 4 differentiation protocols the less *MDM2* amplification was found in NSCs differentiating towards oligodendrocytes. Differentiating oligodendrocytes showed the strongest *MDM2* amplification after 96 h. We found comparable *MDM2* amplification pattern in spontaneously differentiated cells and in differentiating neurons with the highest *MDM2* amplification levels found at 48 h and/or 72 h. Differentiation into the astrocytic lineage showed yet a different pattern with the highest *MDM2* amplification levels at 24 h and 72 h (Figure 2b and Table 1).

Similar to the amplification pattern of *CDK4*, we found amplification for *GINS2* already at 24 h specifically in differentiating oligodendrocytes, differentiating neurons and spontaneously differentiated cells. Subsequently there was a lower *GINS2* copy number at 48 h and at 72 h. At the last time point (96 h) there was again an increased copy number of *GINS2* in differentiating oligodendrocytes while the copy number remained unchanged in spontaneously differentiated cells and differentiating neurons (Table 1). A yet different amplification pattern was found for *TP53*. While spontaneously differentiated cells showed a similarly increased copy number at all 4 time points, differentiating oligodendrocytes showed no copy number increase. Both differentiating neurons and differentiating astrocytes showed the highest copy numbers at the beginning (24 h) of the observed time period (Table 1). Like for the abovementioned genes including *CDK4*, *MDM2*, *GINS2* and *TP53* we found specific pattern of copy number changes for *DDB1*, *EGFR*, *MDM4* and *GFAP* for each of the four differentiating protocols. However, the genes *DDB1*, *EGFR*, *MDM4* and *GFAP* showed not only increased but also decreased copy numbers. As for the increased copy numbers, the time points at which the decrease occurred were different between the affected genes. The copy number of *DDB1* was most decreased in undifferentiated NSCs (Figure 2c). We also found a decrease of the *DDB1* copy number in differentiating oligodendrocytes, specifically at 24 h, 48 h, and 72 h (Table 1). As for *EGFR*, the most decreased copy number was found in differentiating oligodendrocytes, but a decrease in copy numbers of *EGFR* also occurred in differentiating neurons. Likewise, we found decreased copy numbers of *GFAP* in both differentiating oligodendrocytes and differentiating neurons. The overall strongest decrease in copy number was found for *MDM4* with a decrease observed for all 4 differentiation-protocols (Table 1). While we found a decrease in copy number in differentiating oligodendrocytes, neurons and astrocytes, undifferentiated NSCs revealed *MDM4* amplification (Figure 2d).

**Figure 3:**
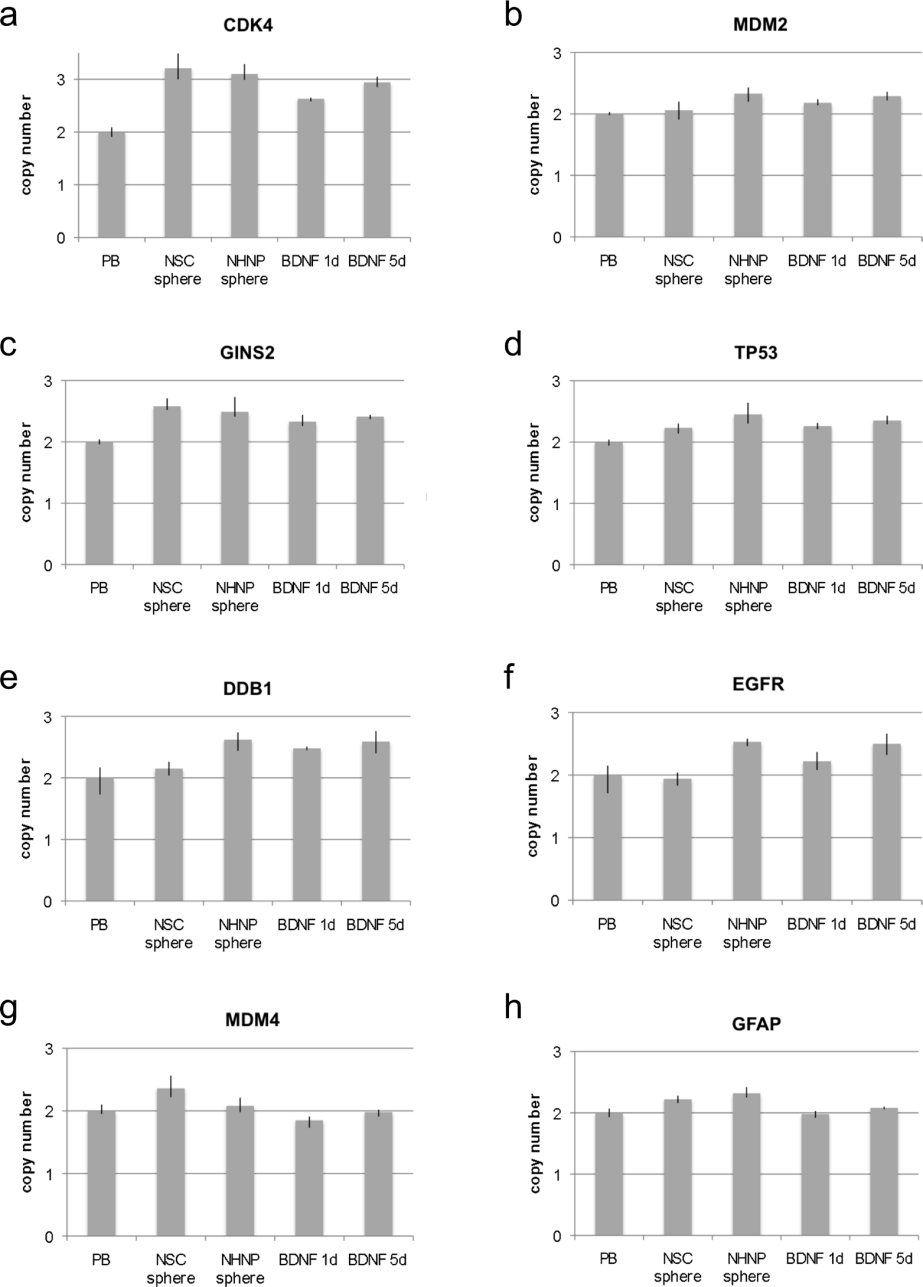
Copy number analysis in neural stem cell spheres and progenitor cell spheres Copy number of *CDK4, MDM2, GINS2*, *TP53 DDB1, EGFR, MDM4* and *GFAP* was analyzed by qPCR using TaqMan copy number assays. *RNaseP* was used as reference gene in the TaqMan assays and DNA from normal blood lymphocytes (PB) served as standard for normal diploid copy number. Copy numbers are shown as mean from four technical replicates with vertical lines indicating the range. Differentiation induction of NHNP sphere cells was either for one day with BDNF (BDNF 1d) or five days with BDNF (BDNF 5d). NSC sphere cells and NHNP sphere cells serve as undifferentiated controls. *CDK4* was amplified with equal copy number in NSC and NHNP sphere cells and after 5 days of differentiation of NHNP sphere cells with BDNF **a.** *MDM2* was amplified in NHNP sphere cells and NHNP sphere cells differentiated for 5 days with BDNF but not in NSC sphere cells **b**. *GINS2* was amplified with equal copy number in NSC sphere cells and NHNP sphere cells and in BDNF differentiated NHNP sphere cells **c**. *TP53* was amplified in NHNP sphere cells and in 5-day differentiation induced NHNP cells but not in NSC sphere cells **d**. *DDB1* was amplified in NHNP sphere cells and differentiation induced NHNP cells but not in NSC sphere cells **e**. *EGFR* was amplified in NHNP sphere cells and in differentiation induced NHNP cells but not in NSC sphere cells **f**. *MDM4* was amplified in NSC sphere cells but not in NHNP sphere cells and differentiation induced NHNP cells **g**. *GFAP* revealed an increased copy number in NSC sphere cells and amplification in NHNP sphere cells but not in differentiation induced NHNP cells **h**.

In addition to adherent growing cells growing as monolayer, cells growing as spheres were also analyzed. In detail we analyzed NSC and human neural progenitor cells (NHNP) (Lonza) both as undifferentiated spheres and NHNP spheres after differentiation induction with BDNF as described previously [5]. Both undifferentiated NSCs and NHNP spheres showed a similar pattern of *CDK4* copy number amplification (Figure 3a). The copy number in both sphere cultures was also higher than in undifferentiated NSCs that were grown as adherent cells. Upon differentiation the copy number of *CDK4* in NHNP spheres was decreased especially after 24 h as compared to the undifferentiated NHNP spheres (Figure 3a). As for *MDM2* we did not detect an increase of the copy number in the NSC spheres. In contrast, we detected *MDM2* amplification in NHNP spheres both in undifferentiated and in BDNF differentiation-induced NHNP spheres as shown in Figure 3b. For *GINS2* we found amplifications in all spheres including NSC spheres, undifferentiated NHNP spheres, and BDNF differentiation-induced NHNP spheres (Figure 3c). Likewise a *TP53* copy number increase occurred in all spheres with the strongest increase in undifferentiated NHNP spheres (Figure 3d). We also found specific pattern of copy number changes for *DDB1, EGFR, MDM4* and *GFAP* in NSC spheres, undifferentiated NHNP spheres, and BDNF differentiation-induced NHNP spheres. A comparable strong copy number decrease as observed for differentiating NSCs did not occur in cells that were grown as spheres. A minimal decrease was found only for *EGFR* in NSC spheres and for *MDM4* in BDNF induced NHNPs (Figure 3f; 3g). By contrast, *DDB1* and *EGFR* show a rather prominent amplification specifically in undifferentiated and in differentiation-induced NHNP spheres (Figure 3e; 3f). *GFAP* revealed a copy number increase in NSC spheres and amplification in undifferentiated NHNP sphere (Figure 3h).

### Amplifications in glioma stem-like cells

**Figure 4:**
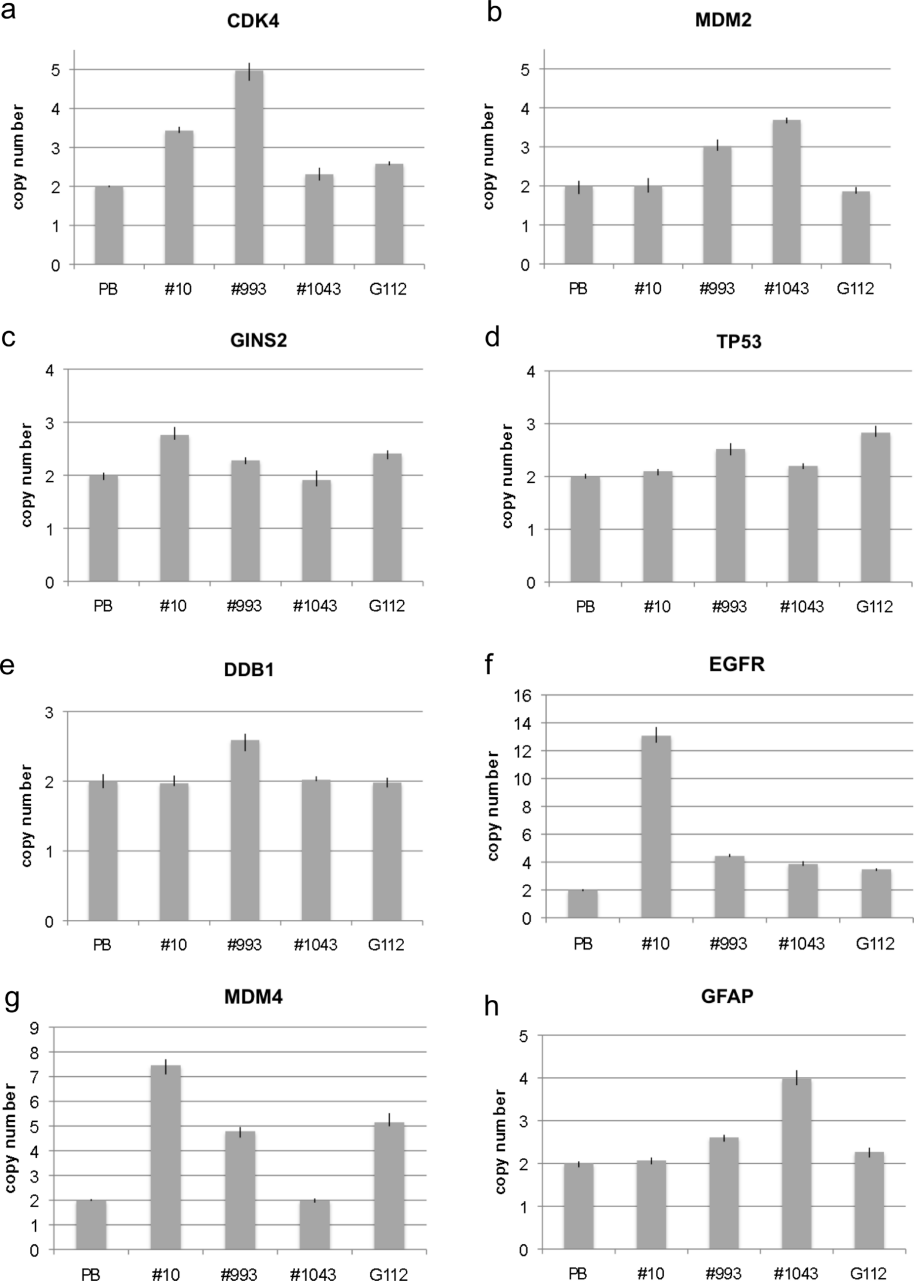
Copy number analysis of eight genes in glioma stem-like cells Copy number of *CDK4* **a**., *MDM2* **b**., *GINS2* **c**., *TP53* **d**., *DDB1* **e**., *EGFR* **f**., *MDM4* **g**. and *GFAP* **h**. was analyzed by qPCR using TaqMan copy number assays. Normal blood lymphocytes (PB) served as standard for normal diploid copy number. Copy numbers are shown as mean from four technical replicates with vertical lines indicating the range.

In addition to neural stem cells and neural progenitor cells and their differentiation towards astrocytes, neurons and oligodendrocytes, we analyzed four glioma stem-like cells (GICs #10, #993, #1043, G112) for amplification. While none of the GICs showed amplification for all genes, all samples showed amplifications for at least 3 genes. In detail, we found *CDK4* amplification in samples GIC#10, GIC #993 and G112 as shown in Figure 4a. *MDM2* amplification was found in GIC#993 and GIC#1043 but not in other lines as shown in Figure 4b. *GINS2* amplification was detected in GIC#10 and G112 as shown in Figure 4c. *TP53* amplification was detected in GIC#993 and G112 (Figure 4d). Interestingly, G112 harbors a mutant *TP53* (mut p53, 273H) as described previously [9–11]. *DDB1* was only amplified in GIC#993 (Figure 4e). *EGFR* amplification was detected in all samples with the highest copy number in GIC#10 (Figure 4f). *MDM4* amplification was present in three samples (GIC#10, GIC#993 and G112) as shown in Figure 4g and *GFAP* amplification was detected in GIC#993 and GIC #1043 (Figure 4h).

### Confirmation of amplification/under-representation by FISH

**Figure 5:**
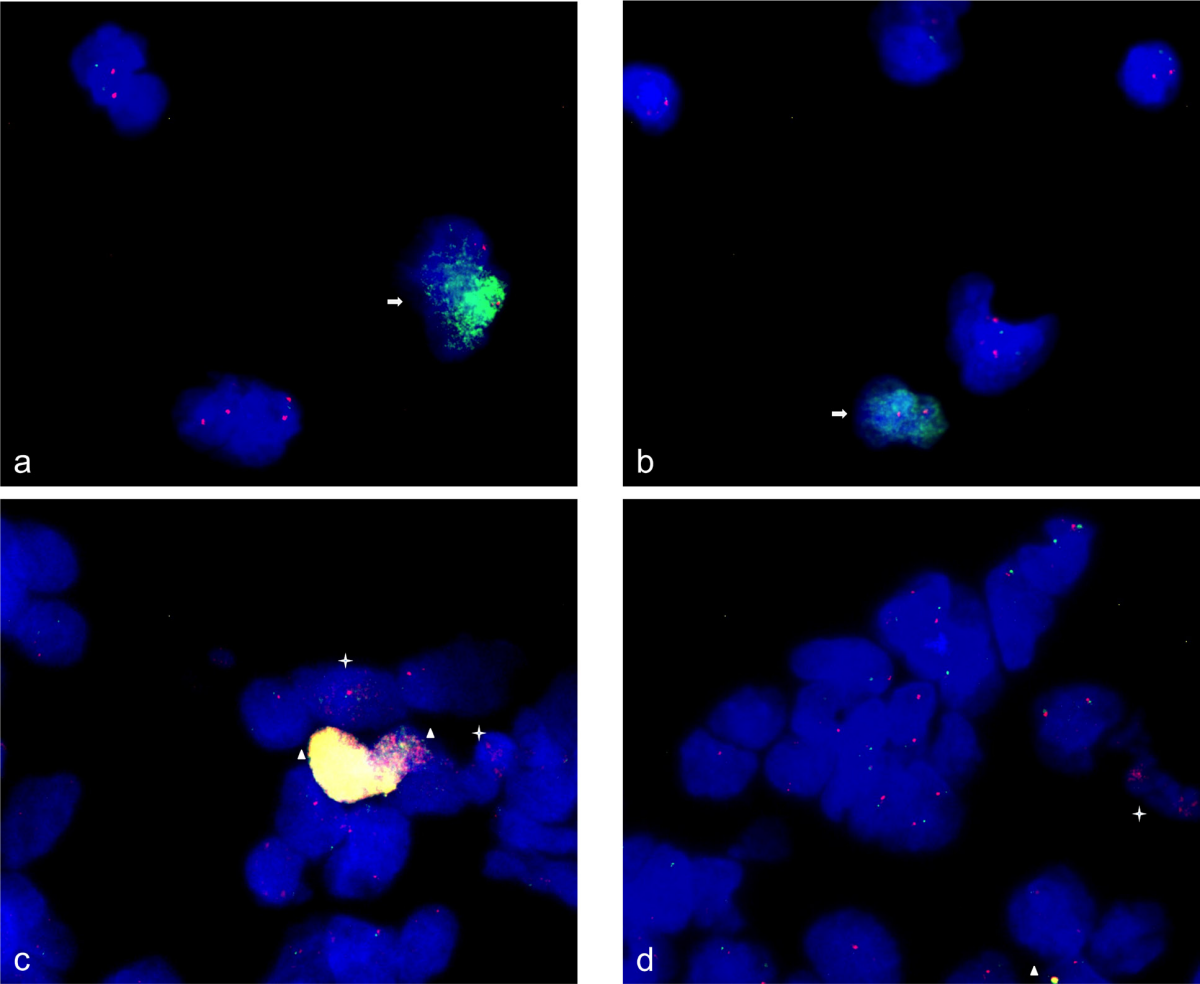
Gene amplifications on chromosomes 12q14-15 and 1q42.13 in human neural stem cells FISH was used to analyze *MDM4* (RP11-433N15) and *CDK4* (RP11-571M6), *MDM2* (RP11-611O2) gene amplification in human neural stem cells. 95% of the neural stem cells revealed two signals for the chromosome alpha-centromere probe (D1Z5) on chromosome 1 (red) and *MDM4* (green) whereas 5% of the neural stem cells revealed two signals for chromosome 1 alpha-centromere and high copy number of hybridization signals for *MDM4* revealing an amplification of *MDM4* **a**., **b**. Similarly 90% of neural stem cells revealed two signals for *CDK4* (red) and *MDM2* (green) with approximately similar fluorescence intensity. In contrast 5% of neural stem cells revealed intense yellow hybridization signals indicating co-amplification of *CDK4* and *MDM2* and 5% of neural stem cells revealed enhanced number of hybridization signals for *CDK4* and two signals for *MDM2* indicating *CDK4* amplification **c**., **d**. Representative cells with amplifications are marked by arrow (*MDM4*), asterisk (*CDK4*) and triangle (*CDK4* and *MDM2*). Nuclei were counterstained with DAPI.

We used fluorescence *in situ* hybridization (FISH) as single cell based technique for confirmation of amplification and under-representation. Human neural stem cells were analyzed undifferentiated, spontaneously differentiated for 24 h and differentiation-induced towards neurons for 48 h. *MDM4* amplification, that was detected by qPCR was confirmed by FISH in undifferentiated human neural stem cells. The *MDM4* amplification was found with a very high number of fluorescence signals in ∼ 5% of neural stem cells. As control we used the centromere probe D1Z5 that maps to chromosome 1 like *MDM4*. The number of fluorescence signals of D1Z5 indicated a normal diploid copy number of this chromosome (Figure 5a and 5b). In addition, we confirmed *CDK4* amplification in 10% of undifferentiated neural stem cells. In 5% of *CDK4*-amplified cells we detected co-amplification of *MDM2* as indicated by very prominent fluorescence signals (Figure 5c and 5d).

**Figure 6:**
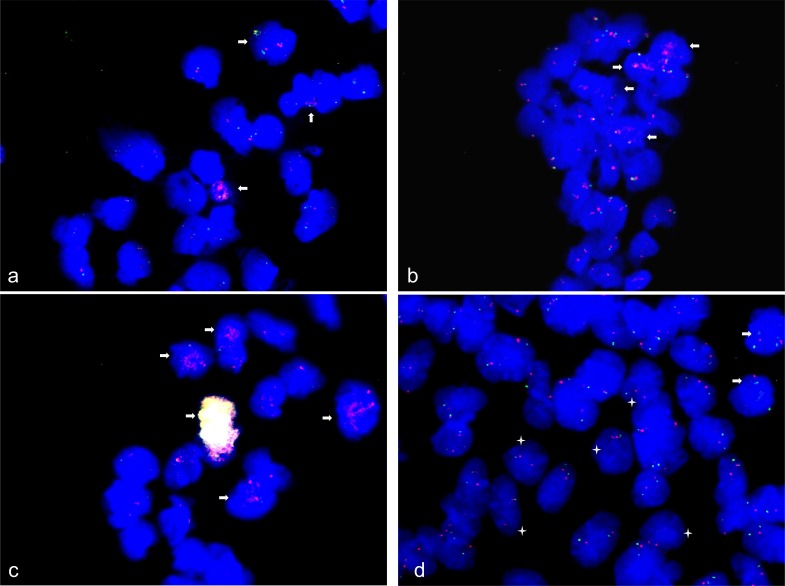
Gene amplification and under-replication in spontaneous and neural differentiation induced human neural stem cells FISH was used to analyze *CDK4* (RP11-571M6), *MDM2* (RP11-611O2), *DDB1* (CTC-820M2) and *MDM4* (RP11-433N15) copy number in human neural stem cells induced to differentiate spontaneous for 24 h and towards neurons for 48 h. *CDK4* (red) and *MDM2* (green) amplifications were detected in 15% of neural stem cells induced to differentiate towards neurons for 48 h **a**. *DDB1* amplification (red) was detected in 10% neural stem cells induced to differentiate towards neurons for 48 h **b**. and remaining cells revealed two copies for *DDB1* and *STX3* control BAC probe (green; RP11-468F15). *CDK4* and *MDM2* high copy co-amplification (yellow/white fluorescence signals) was detected in 1% of cells spontaneously differentiated and in additional 25-30% of cells *CDK4* amplification was detected accompanied with normal *MDM2* copy number (green) **c**. Under-replication of *MDM4* was detected in 20% of cells differentiated spontaneously for 24 h with only one fluorescence signal (green) accompanied with normal copy number of alpha-centromere probe chromosome 1 (red) and amplification of *MDM4* was detected in less than 5% of those cells **d**. Representative cells with amplifications are marked by arrow and cells with under-replication are marked by asterisk. Nuclei were counterstained with DAPI.

FISH also confirmed *CDK4* amplification and *MDM2* amplification in 15% of human neural stem cells that were induced to differentiate towards neurons for 48 h (Figure 6a). Furthermore, FISH confirmed *DDB1* amplification in 10% of human neural stem cells that were induced to differentiate towards neurons for 48 h. Since *DDB1* maps to chromosome 11 we used *STX3* that also maps to chromosome 11 as control. FISH with *STX3* indicated a normal diploid copy number (Figure 6b). *CDK4* and *MDM2* co-amplification was detected in 1% of spontaneously differentiated neural stem cells, by FISH. In addition 25-30% of these cells also revealed *CDK4* amplification but a normal diploid copy number for *MDM2*, which is localized in close vicinity of *CDK4* on chromosome 12 (Figure 6c).

As for spontaneously differentiated neural stem cells FISH identified *MDM4* amplification in 2-5% of the cells and under-representation in 20% of the cells with the latter cases indicated by a single *MDM4* fluorescence signal per cell. FISH with the chromosome 1 centromere probe D1Z5 that was used as reference probe showed two signals indicating a normal diploid status (Figure 6d). FISH also revealed *CDK4* amplification in 5-day BDNF differentiation-induced NHNP cells and *MDM4* under-representation in neural stem cells 48 h induced to differentiate towards oligodendrocytes (Supplementary Figures 1 and 2).

## DISCUSSION

### Amplification and under-replication in context of stem cells

Even undifferentiated neural stem cells harbor multiple gene amplifications as for example *MDM4* that was amplified in neural stem cells but showed a clearly decreased copy number in neural stem cells that were differentiated towards oligodendrocytes, neurons and astrocytes. Similar copy number decreases have been described in the context of gene amplifications in *Drosophila* [12] and most recently in mouse trophoblast cells [4]. Other genes like *CDK4* were amplified not only in undifferentiated neural stem cells and undifferentiated neural progenitor cells but also during the induced differentiation processes of these cells. Notably, the amplification process appears not due to an altered polyploidy since both genes *CDK4* and *MDM2* are mapping on chromosome 12. Chromosome 12 shows a normal copy number in neural stem cell spheres. The copy number changes that are found for chromosome 12 during differentiation are not related to the copy number changes found for *CDK4*. Similar results that confirm amplification independent from polyploidy were found for *TP53* and *GFAP* both localized on chromosome 17, *DDB1* and *STX3* (control probe in FISH experiments) both localized on chromosome 11 and for *MDM4* that revealed either amplification and/or under-replication accompanied with diploid copy number of chromosome 1.

Different amplification patterns were also found between adherently grown neural stem cells and cells that were grown as spheres. A difference of the amplification level was for example found between the genes *GINS2* and *DDB1*. *DDB1* was under-replicated in adherent neural stem cells but had normal copy number in spheres. *GINS2* showed a normal copy number in adherent neural stem cells but was amplified in spheres. Differences in the amplification between spheres formed by neural stem cells or neural progenitor cells were also found for *EGFR*, *TP53*, *MDM2* and *DDB1*.

As for the biological function of the amplified genes, both *GINS2* and *DDB1* are involved in replication. While *GINS2* belongs to the GINS complex that has an essential role in the initiation of DNA replication and for the progression of DNA replication forks [13], *DDB1* is involved in replication control through ubiquitination and degradation of CDT1 [14]. *TP53* and *DDB1* are involved in DNA damage response. *MDM2* and *MDM4* are p53-regulating proteins with complementary but distinct roles in the regulation of TP53 activities. Both contain a p53-binding domain and are thought to be involved in suppression of TP53 transactivation and apoptotic function. Since the *TP53* amplification was predominantly detected in neural progenitor cells and in neural stem cells differentiating towards neurons and astrocytes or towards neural and glial cells, *TP53* amplification is likely to be an attribute of differentiating cells. This observation is consistent with the observation by Meletis and colleagues reporting that TP53 suppresses self-renewal in adult neural stem cells [15]. Furthermore, *TP53* up-regulation contributes to maintaining genome integrity in progenitor cells during differentiation steps that are prone to genome instability. Interestingly, we found a very high amplification level for the *GFAP* gene in neural stem cells and neural progenitor cells despite their lack of GFAP immunoreactivity for common anti-GFAP antibodies. Vice versa, no *GFAP* amplification was detected in differentiating neural stem or progenitor cells, which are characterized by increased expression of GFAP at the onset of differentiation. One possible explanation is that *GFAP* amplification may be associated with alternative splicing resulting in the expression of GFAP isoforms that may not be recognized by anti-GFAP antibodies commonly used in histological analyses. For example, one of the 8 known GFAP isoforms, GFAP∂ is highly expressed in radial glia and subventricular zone progenitors [16] but cannot be detected by commonly used anti-GFAP antibodies binding to the C-terminus which is different between GFAP∂ and the most abundant isoform GFAPα.

### Confirmation of amplification and/or under-replication

There are limitations to all approaches tailored to detect copy number alterations including amplifications, polyploidy, deletions and under-replication. Both array-CGH and NGS allow to obtaining an overview on copy number changes. Standard second-generation sequencing is unlikely to unravel the amplification patterns when only a fraction of the analyzed cells carry amplifications. As for the array analysis, a threshold below log_2_ 0.8 was frequently used to define deletions and a threshold higher log_2_ 1.2 to define amplifications. While these thresholds were valuable for uniform cell populations they are of limited use for heterogeneous cell populations especially for analyzing small sub-populations carrying copy number alteration. For example our previous whole genome tiling array-CGH analysis detected *CDK4* amplifications in 5-days BDNF differentiated NHNP cells but not in 1-day BDNF differentiated NHNP cells [5]. Here, we choose threshold settings for qPCR analysis to identify copy number alterations that are limited to small sub-populations in heterogeneous cell populations. Comparable thresholds of >2.31 and >2.5 were used in studies on *TERC* gene amplification in chronic myeloid leukemia and *EGFR* copy number in metastatic lung cancer [17, 18]. In addition, we used fluorescence *in situ* hybridization to detect copy number alterations that occur in very few cells. For example, *MDM4* under-representation in 24 h spontaneously differentiated hNSC cells was not detected by qPCR analysis even using our threshold settings but by FISH, which revealed loss of one copy in 20-25% of the cells.

Notably, we did not detect copy number decreases by qPCR in sphere cells. This may be due to the fact that sphere cells were only gradually exposed to differentiation inducing conditions. Depending on sphere size the relative number of cells with copy number changes is lower than in cell culture. While gene amplifications with a high copy number increase to more than 50 copies are still detectable in spheres, copy number decreases with only one copy lost per cell, go very likely undetected in spheres. The potential of alternative whole genome sequencing to detect copy number changes is controversially discussed [19]. Tattini and co-workers indicated third generation sequencing as potential solution, which in our opinion may be ideally combined with single cell sequencing.

### Amplification in context of glioma cells

*EGFR* is amplified in almost 50% of glioblastoma and 20% of anaplastic oligodendroglioma. Previously, we did not detected *EGFR* amplifications in undifferentiated NHNP sphere cells by whole genome tiling array-CGH analysis only showing log_2_-ratio values that did not reach the threshold for amplification [5]. Likewise, array-CGH did not detect EGFR amplifications in human and mouse neural progenitor cells neither in a differentiated nor in a not-differentiated state. By contrast, other genes including *CDK4* and *MDM4* frequently amplified in glioblastoma were also amplified in human neural progenitor cells during differentiation [5]. Using TaqMan PCR analysis we now show *EGFR* amplifications in human neural progenitor cells and in human neural progenitor cells during differentiation. Interestingly we did also detect *EGFR* amplification in neural stem cells differentiating towards the astrocytic lineage. Our array-based amplification analyses warrant a more detailed analysis of gene and chromosome regions to get a deeper insight in gene amplification pattern in stem and progenitor cells.

The amplification data of stem and progenitor cells and the amplification data obtained for glioma stem-like cells, can help to define cell populations at the origin of the glioma stem-like samples. Since *MDM4* was only amplified in neural stem cells, neural stem cell are likely to be at the origin of the glioma stem-like cells #10, #993 and G112. *EGFR* amplification in undifferentiated and differentiating NHNP cells, point to neural progenitor cells at the originating of glioma stem-like cell samples. *DDB1* amplification was only present in NHNP cells and in neural stem cells that were differentiation induced towards astrocytes. *DDB1* copy number increase was present during beginning differentiation of neural stem cells towards neurons (24-48h). During further differentiation of neural stem cells towards oligodendrocytes for 72-96h, copy number increased from under-replicated state to normal copy number. These data indicate a more differentiated cell type as originating cell in glioma stem-like cell #993 derived from oligodendroglioma. *GFAP* amplification was only detected in neural stem cells and neural progenitor cells but not in differentiation-induced progeny of neural stem and progenitor cells. These data indicate a neural stem and neural progenitor cell at the origin of glioma stem-like samples #993 and #1043. In conclusion, gene amplifications specifically gene amplifications of *DDB1*, *EGFR*, *MDM4* and *GFAP* can provide information about the cell types and the degree of heterogeneity at the origin of glioma stem-like cells. *CDK4*, *MDM2*, *GINS2* and *TP53* amplifications appeared less or not suitable to narrow down the originating cell composition. It is possible that the tumor reuse the same amplification pattern that is physiologically defined to normal stem cells. While this information can help to learn more about the origin of glioma it will not have immediate impact on new clinical treatment strategies of glioma.

### Amplification maintained throughout stem cell divisions

Interestingly, *CDK4* amplification appears to be rather frequent with amplification detected in all investigated samples. Notably, although fluctuations in *CDK4* copy number were found during differentiation, the *CDK4* amplification was found to be a persistent phenomenon. During differentiation of neural stem cells towards oligodendrocytes, amplification of *CDK4* even increased after four days. Fluorescence *in situ* hybridization of neural progenitor cells indicated *CDK4* amplifications in a limited number of neural progenitor cells with asymmetrical cell division (Supplementary Figure 1). The maintained *CDK4* amplification in only few cells suggests that *CDK4* amplification contributes to the overall regenerative capacity of the whole population. Our results support the idea that basic amplification level of *CDK4* is conserved in neural stem cells, in neural progenitor cells, and in their differentiated progeny. It is tempting to hypothesize that *CDK4* amplification is important to maintain proliferation and self-renewal propensity not only in neural stem cells but also in their differentiated progenies. Notably, the highest level of *CDK4* amplification was found under oligodendrocyte-inducing conditions (Table 1). Furthermore, although the increase in *CDK4* amplification was detected under all conditions tested at 1 day after differentiation induction, its magnitude was most persistent under oligodendrocyte-inducing condition. In this regard, it is noteworthy that *CDK4* plays an important role in the maintenance of self-renewal in NG2+ progenitors that comprise a principal population of proliferation-capable cells for oligodendrocytes in the olfactory bulb [20, 21]. Further testing is required to clarify the relationship between *CDK4* amplification and self-renewal capacity.

## MATERIALS AND METHODS

### Cell culture and differentiation

NHNP cells were grown and differentiated as described previously [5]. GIBCO Human neural stem cells (H9 hESC-derived) further named NSC, were cultured on CELLStart^TM^-coated culture ware with complete StemPro NSC SFM medium as described in the manufacturers’ instructions. Neural stem cell sphere cultures were grown in uncoated culture ware.

For spontaneously differentiation of NSCs, these cells were plated at 2.5×10^4^ cells/cm^2^ on CELLStart tissue culture plates in StemPro NSC SFM medium without bFGF and EGF.

For differentiation towards oligodendrocytes NSC were plated at 2.5×10^4^ cells/cm^2^ on poly-L-ornithine/laminin coated tissue culture plates with Neurobasal Medium (supplemented with B-27 serum-free supplement, GlutaMAX and 30ng/ml T3).

For differentiation towards astrocytes NSC were plated at 2.5×10^4^ cells/cm^2^ on Geltrex matrix coated tissue culture plates with D-MEM (supplemented with N-2 supplement, GlutaMAX and 1%FBS).

For differentiation towards neurons NSC cells were plated at 2.5×10^4^ cells/cm^2^ on poly-L-ornithine coated tissue culture plates with Neurobasal Medium (supplemented with B-27 serum-free supplement and GlutaMAX).

Glioma stem-like cells (GIC) were isolated from surgical specimens and characterized as described previously [11, 22].

### DNA isolation

Cells were harvested and DNA was isolated using chlorofom/NaCl method. In brief cell pellet was digested with Proteinase K at 55°C over night (> 12h) and chloroform extracted for 1h at room temperature.

### QPCR analysis

TaqMan Copy Number Assays for genes *CDK4* (Hs00957586_cn), *MDM2* (Hs00181272_cn), *GINS2* (Hs05472641_cn), *TP53* (Hs05506931_cn), *DDB1* (Hs07226265_cn), *EGFR* (Hs01463609_cn), *MDM4* (Hs05784087_cn), *GFAP* (Hs01144882_cn) were performed following manufacturers instructions. We used the *RNaseP* TaqMan Copy Number reference assay for relative quantitation of copy number of target genes. DNA from human normal blood lymphocytes (PB) was used as control standard for normal diploid copy number.

TaqMan assays were run in four technical replicates and results were analyzed using StepOne^TM^ Software v2.0 and CopyCaller^TM^ software.

### Fluorescence *in situ* hybridization

BAC clones were taken either from the RP11 Human Male BAC library, that was generated by the BACPAC Resource Center (BPRC) at the Children's Hospital Oakland Research Institute by Kazutoyo Osoegawa [23] or from RZPD made available through SourceBioSciences, Germany. BAC-DNA (1μg) was either labeled with Alexa-488-dCTP, with Alexa-555-dCTP or with Alexa-594-dCTP using the FISHTag DNA labeling Kit according to the manufacturer's instructions. Differentially labeled probe DNAs (60 ng) were precipitated in the presence of human Cot-1 DNA. Samples were resuspended in hybridization mix (50% formamide, 2xSSPE, 10% dextrane sulphate and 4% SDS). Alpha-centromere probe from chromosome 1 (D1Z5) was added to the resuspended probe mix.

Differentiating human neural stem cells and undifferentiated neural stem cells were grown on glass slides with appropriate coating and fixed in ice-cold methanol for 20 minutes. Slides were washed in PBS for 5 minutes and treated with 0.02% Tween-20 for 3 minutes. Slides were RNase treated (100 μg/ml RNaseA in 2x SSC) for 1h at 37°C and pepsin treated (0.005% in 0.01 M HCl at 37°C) for 10 minutes. Postfixation was performed using 1% formaldehyde/1x PBS for 10 minutes at room temperature. Hybridization and posthybridization washes were as described previously [6].

## SUPPLEMENTARY MATERIALS FIGURES



## Abbreviations

array-CGH: array comparative genomic hybridization
BDNF: brain-derived neurotrophic factor
CDK4: cyclin-dependent kinase 4
CDT1: Chromatin Licensing And DNA Replication Factor 1
DDB1: DNA-damage-binding protein 1
EGFR: epidermal growth factor receptor
FISH: fluorescence *in situ* hybridization
GFAP: Glial Fibrillary Acidic Protein
GIC: glioma stem-like cell
GINS2: *go-ichi-ni-san 2*
hESC: human embryonic stem cell
MDM2: mouse double minute 2
MDM4: mouse double minute 4
NG2+: nerve/glial antigen 2 positive
NHNP: normal human neural progenitor
NSC: neural stem cell
PB: peripheral blood
qPCR: quantitative polymerase chain reaction
SFM: serum free medium
