# Supplementary material for: Specific amplifications and copy number decreases during human neural stem cells differentiation towards astrocytes, neurons and oligodendrocytes
Source: Oncotarget. 2017 Mar 7;8(16):25872–84. doi: 10.18632/oncotarget.15980 (PMC5432223; doi:10.18632/oncotarget.15980)
Supplement: Supplementary file 1 [file oncotarget-08-25872-s001.pdf]

Specific amplifications and copy number decreases during human neural stem cells differentiation towards astrocytes, neurons and oligodendrocytes

Supplementary Materials

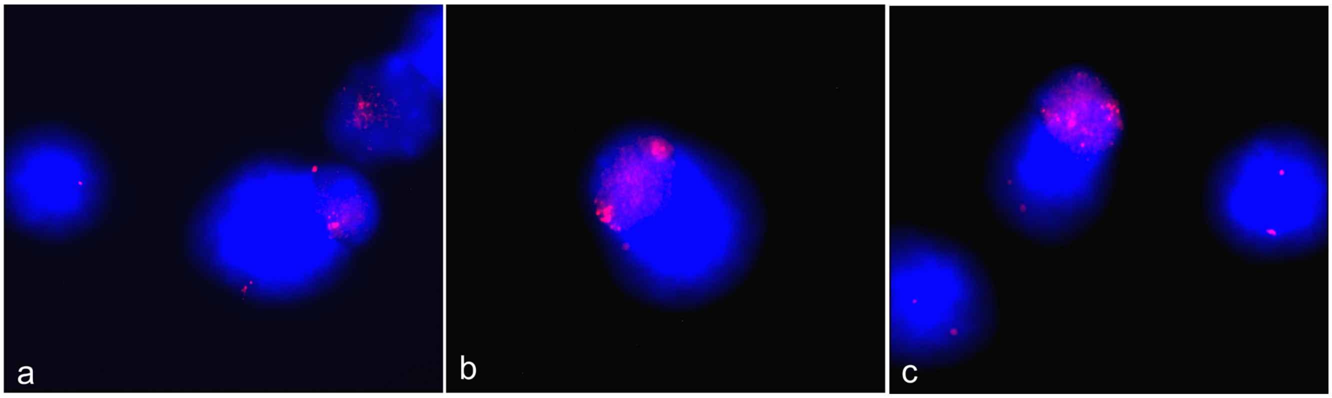

Supplementary Figure 1: Additional FISH results on 5-day differentiated NHNP cells. Representative fluorescence *in situ* hybridization results indicating *CDK4* amplification using *CDK4* (RP11-571M6) (red) on 5-day differentiated NHNP cells (a-c). Nuclei were counterstained with DAPI.

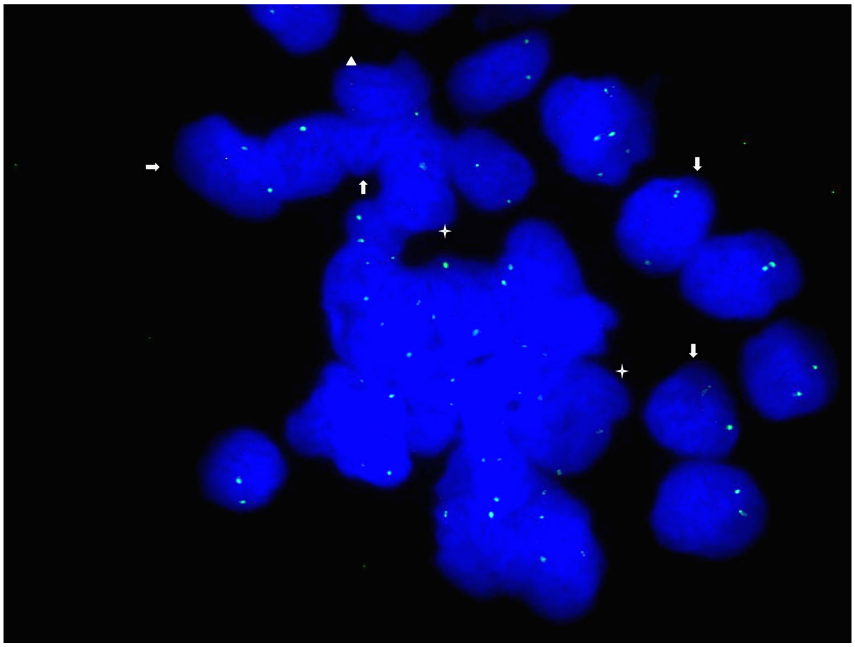

Supplementary Figure 2: Additional FISH results on neural stem cells differentiated towards oligodendrocytes Representative fluorescence in situ hybridization results indicating *MDM4* under-replication using *MDM4* (RP11-433N15) (green) on neural stem cells differentiated for 48h towards oligodendrocytes. Cells without a detectable fluorescence signal for *MDM4* were marked by star. Cell with two faint fluorescence signals for *MDM4* was marked by triangle. Cells with bright and faint fluorescence signal for *MDM4* were marked by arrow. Nuclei were counterstained with DAPI.
